# PRMT3‐Mediated Arginine Methylation of METTL14 Promotes Malignant Progression and Treatment Resistance in Endometrial Carcinoma

**DOI:** 10.1002/advs.202303812

**Published:** 2023-11-16

**Authors:** Yiru Wang, Can Wang, Xue Guan, Ying Ma, Shijie Zhang, Fei Li, Yue Yin, Zhenxing Sun, Xiuwei Chen, Hang Yin

**Affiliations:** ^1^ Department of Gynecologic Oncology Harbin Medical University Cancer Hospital Harbin Heilongjiang 150000 China; ^2^ Animal Laboratory Center The Second Affiliated Hospital of Harbin Medical University Harbin Heilongjiang 150000 China; ^3^ Department of Radiation Oncology Harbin Medical University Cancer Hospital Harbin Heilongjiang 150000 China; ^4^ NHC Key Laboratory of Molecular Probes and Targeted Diagnosis and Therapy Harbin Medical University Harbin Heilongjiang 150000 China; ^5^ Department of Health Technology and Informatics Hong Kong Polytechnic University Hung Hom Kowloon Hong Kong 27665111 China

**Keywords:** endometrial cancer, ferroptosis, METTL14, PRMT3, therapeutic resistance

## Abstract

Protein arginine methyltransferase (PRMT) plays essential roles in tumor initiation and progression, but its underlying mechanisms in the treatment sensitivity of endometrial cancer (EC) remain unclear and warrant further investigation. Here, a comprehensive analysis of the Cancer Genome Atlas database and Clinical Proteomic Tumor Analysis Consortium database identifies that PRMT3 plays an important role in EC. Specifically, further experiments show that PRMT3 inhibition enhances the susceptibility of EC cells to ferroptosis. Mechanistically, PRMT3 interacts with Methyltransferase 14 (METTL14) and is involved in its arginine methylation. In addition, PRMT3 inhibition‐mediated METTL14 overexpression promotes methylation modification via an m^6^A‐YTHDF2‐dependent mechanism, reducing Glutathione peroxidase 4 (GPX4) mRNA stability, increasing lipid peroxidation levels, and accelerating ferroptosis. Notably, combined PRMT3 blockade and anti‐PD‐1 therapy display more potent antitumor effects by accelerating ferroptosis in cell‐derived xenograft models. The specific PRMT3 inhibitor SGC707 exerts the same immunotherapeutic sensitizing effect in a patient‐derived xenograft model. Notably, blocking PRMT3 improves tumor suppression in response to cisplatin and radiation therapy. Altogether, this work demonstrates that PRMT3 depletion is a promising target for EC.

## Graphical abstract

PRMT plays a crucial role in tumor progression through unclear mechanisms. This study shows that PRMT3 depletion fails to bind and arginine methylate METTL14, which downregulates GPX4 in an m^6^A‐dependent manner, and its m^6^A site consensus sequence is recognized and bound by YTHDF2, ultimately inhibiting cellular resistance to ferroptosis and leading to therapeutic sensitization in endometrial cancer.

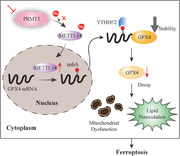

## 1 Introduction

Endometrial cancer (EC) is a highly prevalent form of gynecological malignancy, accounting for over 4 10 000 new diagnoses and 9 7000 deaths globally in 2020.^[^ ^1^, ^2^ ^]^ Effective and specific biomarkers for EC require in‐depth investigation given their uncertainty and elusiveness. Currently, the main treatment options for EC patients include surgical resection, radiotherapy, and chemotherapy. However, there are still 15–20% cases of relapse or even progression after treatment.^[^ ^3^ ^]^ Immune checkpoint inhibitors (ICIs), particularly anti‐PD‐1, are a landmark cancer treatment modality, but only a minority of patients will benefit from it in the long term.^[^ ^4^, ^5^ ^]^ The KEYNOTE‐28 study indicated that pembrolizumab therapy provided a 13% (3/24) objective response rate in PD‐L1‐positive EC patients.^[^ ^5^, ^6^ ^]^ As a result, it remains critical to identify novel biomarkers and explore their mechanisms.

Protein arginine methylation is a crucial posttranslational modification undertaken by protein arginine methyltransferases (PRMTs), the enzymes that catalyze arginine residues in a monomethylated or dimethylated manner.^[^ ^7^, ^8^, ^9^ ^]^ PRMT3, a type I PRMT that mainly catalyzes the asymmetric dimethylation of arginine residues, plays an integral role in controlling carcinogenesis by participating in processes including cellular metabolism and gene regulation.^[^ ^10^ ^]^ To date, PRMT3 has been reported to be upregulated in malignant tumors, such as pancreatic cancer, colorectal cancer, and hepatocellular carcinoma.^[^ ^11^, ^12^, ^13^ ^]^ PRMT3‐mediated methylation of HIF1α could cause abnormal angiogenesis to mediate colorectal tumorigenesis and could increase glycolysis to facilitate glioblastoma cell proliferation.^[^ ^12^, ^14^ ^]^ Additionally, PRMT3 was found to contribute to the induction of apoptosis in breast cancer cells.^[^ ^15^ ^]^ However, whether PRMT3 affects the sensitivity of EC to treatment remains uncertain.

Ferroptosis is an iron‐dependent form of oxidative stress cell death and plays a prominent role in various biological processes.^[^ ^16^ ^]^ Tumor cells generally require more abundant iron and seem susceptible to ferroptosis.^[^ ^17^ ^]^ Intriguingly, tumor cells with treatment resistance are extremely sensitive to ferroptosis inducers.^[^ ^18^ ^]^ Emerging evidence has shown that ferroptosis induction is a promising therapeutic strategy with immense potential.^[^ ^19^ ^]^ Glutathione peroxidase 4 (GPX4), an enzyme capable of eliminating phospholipid hydroperoxides pertinently and efficiently, facilitates the inhibition of the ferroptosis process.^[^ ^20^ ^]^ GPX4 might be considered a focal point to explore the unknown regulatory factors of ferroptosis sensitivity in tumors.^[^ ^16^ ^]^ In addition, immunotherapy has been reported to induce the secretion of IFN‐γ from CD8+ T cells and ultimately drive the onset of ferroptosis in cancer cells.^[^ ^21^ ^]^ Ferroptosis may be closely linked to immunotherapy resistance, and therefore, the search for key targets and prognostic markers of ferroptosis resistance is expected to overcome immunotherapy resistance and improve patient survival.

Many researches have shown that N6‐methyladenosine (m^6^A) plays an important role in cancer.^[^ ^22^ ^]^ m^6^A is transferred through the m^6^A consensus site on mRNA, causing it to be modified by methylation.^[^ ^23^ ^]^ Recently, our group reported that m^6^A methylation‐mediated RMRP modulates the TGFBR1/SMAD2/SMAD3 pathway, thus promoting non‐small cell lung cancer progression.^[^ ^24^ ^]^ Methyltransferase 14 (METTL14) attenuates the proliferation and metastasis of colorectal tumors by downregulating the lncRNA XIST.^[^ ^25^ ^]^ The inhibitory effect of METTL14 on the self‐renewal capacity of bladder tumor‐initiating cells was also emphasized.^[^ ^26^ ^]^ Another methyltransferase, METTL3 mediates tumor‐associated neutrophil infiltration in a synergistic manner with YTHDF2 to prevent papillary thyroid carcinoma.^[^ ^27^ ^]^ However, the value of PRMT and methyltransferase in terms of ferroptosis in EC and whether they might improve therapeutic efficacy are unclear.

Herein, we determined that PRMT3 depletion enhanced the sensitivity of EC cells to ferroptosis. Mechanistically, PRMT3 mediated the arginine methylation of METTL14 and acted as a repressor of the METTL14/YTHDF2 m^6^A axis directing GPX4 mRNA degradation. Knockdown of PRMT3 increased lipid peroxidation levels in cells and accelerated tumor ferroptosis, all of which inhibited resistance to ferroptosis in EC. Clinical data further supported the high expression of PRMT3, the low expression of METTL14, and the negative correlation between the two in EC patients. Furthermore, the therapeutic superiority of PRMT3 inhibition in conjunction with classical therapies was corroborated by cell‐derived xenograft (CDX) and patient‐derived xenograft (PDX) tumor model results. Together, our study demonstrated the importance of PRMT3 in ferroptosis, explored the applicability of PRMT3 inhibition in combination with immunotherapy, radiotherapy, or chemotherapy, and indicated that PRMT3 may serve as a promising target for enhancing antitumor efficacy.

## 2 Results

### 2.1 PRMT3 Is a Critical Upregulated Biomarker in EC

**Figure 1:**
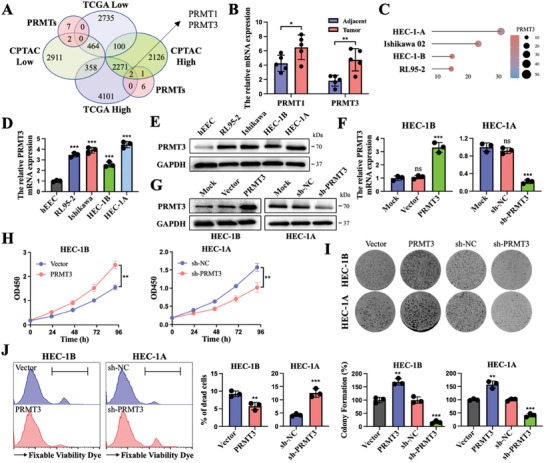
PRMT3 is a critical upregulated biomarker in EC and stimulates cell death. A) Schematic illustration showing the overlapping PRMTs within the UCEC dataset in TCGA and CPTAC. B) The expression of PRMT1 and PRMT3 in 5 clinical EC tissues and matched adjacent normal tissues. C) Expression of PRMT3 mRNA in EC cell lines in the CCLE dataset. The size and color of the dots represent the level of expression. D,E) mRNA expression (D) and protein expression (E) of PRMT3 in EC cell lines (HEC‐1A, HEC‐1B, Ishikawa, and RL95‐2) compared with normal endometrial epithelial cells (hEEC). F,G) mRNA (F) and protein (G) levels of PRMT3 in HEC‐1B cells transfected with PRMT3 or vector and those in HEC‐1A cells transfected with sh‐PRMT3 or sh‐NC. H) CCK‐8 assays of HEC‐1B cells transfected with PRMT3 or vector and HEC‐1A cells transfected with sh‐PRMT3 or sh‐NC. I) Representative images (up) and quantification (down) of colony formation assays in HEC‐1B cells and HEC‐1A cells transfected with control, overexpressed PRMT3 and sh‐PRMT3. J) Fixable viability staining assays in HEC‐1B cells transfected with PRMT3 or vector and HEC‐1A cells transfected with sh‐PRMT3 or sh‐NC. ns, no significance; ^*^ *p* < 0.05, ^**^ *p* < 0.01, and ^***^ *p* < 0.001. Data presented as mean ± SD from at least three independent experiments.

To identify PRMTs that are critical in EC, we performed differential analysis in the UCEC datasets from the TCGA database and the CPTAC database and discovered that PRMT1 and PRMT3 were elevated in EC tissues in the overlapping results (**Figure** **1**A). qRT‒PCR assays confirmed that both PRMT1 expression and PRMT3 expression were higher in 5 clinical EC tissue samples than in the matched paracarcinoma tissues (Figure 1B). Since PRMT1 has been more extensively studied in cancer than PRMT3, we focused on exploring the function of PRMT3 (Figure S1A, Supporting Information). In addition, PRMT3 was upregulated in EC cells compared to normal endometrial epithelial cells. PRMT3 expression was highest in HEC‐1A cells and lowest in HEC‐1B cells among the EC cell lines (Figure 1C–E).

To further determine the role of PRMT3, we established stable HEC‐1B‐Lv‐PRMT3 and HEC‐1A‐Lv‐shPRMT3 cells by lentivirus transfection. Stable genetic transfer effectiveness was validated by western blot and qRT‒PCR (Figure 1F–G; Figure S1B, Supporting Information). Up‐regulation of PRMT3 increased the cell viability of HEC‐1B cells compared to the control, whereas cell viability was reduced in PRMT3‐deficient HEC‐1A cells (Figure 1H). Besides, PRMT3 overexpression increased the colony‐forming ability of HEC‐1B cells and HEC‐1A cells in comparison to the control. In contrast, the opposite trend was observed upon PRMT3 depletion in HEC‐1B cells and HEC‐1A cells (Figure 1I). Furthermore, PRMT3 overexpression significantly decreased the proportion of cell death, whereas knockdown of PRMT3 increased this proportion (Figure 1J). The above findings implied that PRMT3 expression was upregulated in EC and may play an important role in EC progression.

### 2.2 PRMT3 Depletion Leads to Ferroptosis Susceptibility in EC

**Figure 2:**
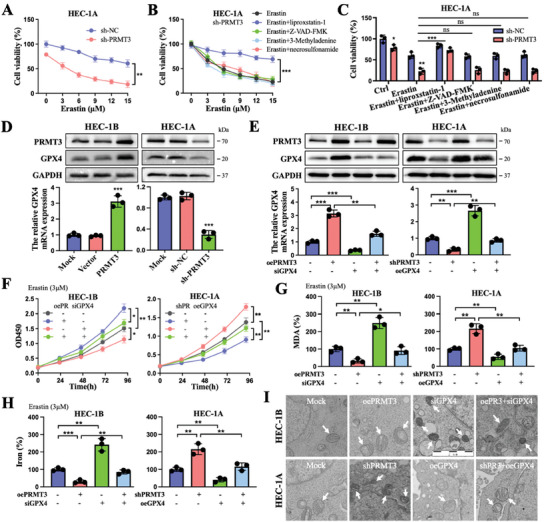
PRMT3 depletion leads to ferroptosis susceptibility in EC and GPX4 reverses its role. A) Cell viability of HEC‐1A cells expressing sh‐PRMT3 or sh‐NC treated with different concentrations of erastin for 48 h. B) Cell viability of HEC‐1A cells transfected with sh‐PRMT3 and treated with erastin in the absence or presence of liproxstatin‐1 (400 nm), ZVAD‐FMK (10 µm), 3‐methyladenine (1 mm) or necrosulfonamide (10 µm) for 48 h. C) Cell viability of HEC‐1A cells transfected with sh‐PRMT3 or sh‐NC and treated with erastin (6 µm) in the absence or presence of liproxstatin‐1 (400 nm), ZVAD‐FMK (10 µm), 3‐methyladenine (1 mm) or necrosulfonamide (10 µm) for 48 h. D) Protein and mRNA levels of GPX4 in HEC‐1B cells transfected with PRMT3 or vector and HEC‐1A cells transfected with sh‐PRMT3 or sh‐NC. E) Protein and mRNA levels of GPX4 in HEC‐1B cells transfected with PRMT3 or siGPX4 and HEC‐1A cells transfected with sh‐PRMT3 or GPX4. F–H) Cell viability (F), relative MDA level (G), and relative ferrous ion level (H) of HEC‐1B cells transfected with PRMT3 or siGPX4 and HEC‐1A cells transfected with sh‐PRMT3 or GPX4 and treated with erastin (3 µm). I) Mitochondrial changes in different groups of cells using transmission electron microscopy (white arrow indicates mitochondria), Scale bars: 2 µm. ns, no significance; ^*^ *p* < 0.05, ^**^ *p* < 0.01, and ^***^ *p* < 0.001. Data presented as mean ± SD from at least three independent experiments.

To investigate the potential function of PRMT3, we explored genes coexpressed with PRMT3 in the TCGA database (cor > 0.4, *p* < 0.01). The KEGG result showed that PRMT3 was significantly enriched in the ferroptosis pathway (Figure S1C, Supporting Information). Insensitivity to cell death, which confers partial tumor resistance to therapeutic modalities, is one of the crucial properties of cancer. Treatment of PRMT3‐downregulated or control EC cells with different concentrations of ferroptosis inducers showed that PRMT3 deletion inhibited resistance to elastin‐induced cell death (**Figure** **2**A). Cells in both the PRMT3‐blocked group and erastin‐treated groups showed some degree of ferroptosis; however, the degree of ferroptosis was significantly increased in the cotreated cells (Figure S1D, Supporting Information). Additionally, this erastin‐induced cell death facilitated by PRMT3 knockdown was rescued by the ferroptosis inhibitor liproxstatin‐1 but not by inhibitors of apoptosis (Z‐VAD‐FMK), necrosis (necrosulfonamide), or autophagy (3‐methyladenine) (Figure 2B,C). These results reveal that the absence of PRMT3 enhances the inhibitory effect of erastin treatment by increasing intracellular ferroptosis.

As GPX4 is a distinguishing marker of the ferroptosis process, further experiments were carried out. The overexpression of PRMT3 promoted GPX4 expression at both the protein and mRNA levels (Figure 2D). Notably, GPX4 upregulation or suppression had no influence on PRMT3 expression. PRMT3 was identified to play a regulatory role as an upstream factor of GPX4 (Figure 2E; Figure S1E, Supporting Information). Next, we sought to investigate the effect of PRMT3 and GPX4 on ferroptosis sensitivity in EC. CCK8 assays demonstrated enhanced cell viability in PRMT3‐overexpressing cells and GPX4‐overexpressing cells, whereas the opposite effects were observed in PRMT3‐deficient cells and GPX4‐deficient cells. GPX4 inhibition or upregulation partially reversed the changes in cell activity induced by PRMT3 overexpression or knockdown, respectively (Figure 2F). Subsequently, malondialdehyde (MDA) and ferrous iron (Fe^2+^) levels were reduced in PRMT3‐overexpressing cells and GPX4‐overexpressing cells and increased in PRMT3‐deleted cells and GPX4 knockdown cells. The attenuated MDA and Fe^2+^ levels caused by PRMT3 overexpression or the elevated trend caused by PRMT3 deletion could be rescued by GPX4 inhibition or overexpression, respectively (Figure 2G,H). Representative transmission electron microscopy images showed that PRMT3‐deficient cells and GPX4‐deficient cells exhibited smaller mitochondria with enhanced membrane density, which is characteristic of ferroptosis. The down‐ or upregulation of GPX4 partially altered the degree of intracellular ferroptosis induced by the up‐ or downregulation of PRMT3, respectively (Figure 2I). Collectively, these data suggested that silencing PRMT3 could inhibit the resistance of cancer cells to ferroptosis by suppressing GPX4 expression in EC.

### 2.3 PRMT3 Arginine Methylates METTL14 to Negatively Regulate Its Protein Expression

**Figure 3:**
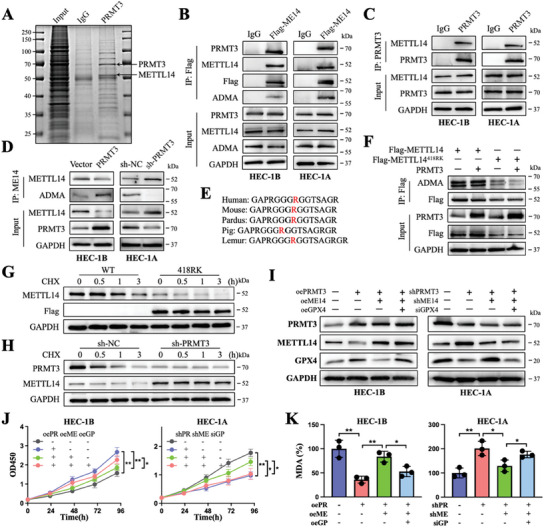
PRMT3 promotes arginine methylation of METTL14, forming the PRMT3‐METTL14‐GPX4 regulatory axis. A) Mass spectrometry analysis of the protein complex binding to PRMT3 from HEC‐1A cells. Blue staining shows the location of PRMT3 and its associated protein METTL14. B) Flag‐tagged METTL14 proteins were immunoprecipitated and subjected to western blot to detect their interaction with PRMT3 and the ADMA signal. C) PRMT3 proteins were immunoprecipitated and subjected to western blot to detect their interaction with METTL14. D) METTL14 proteins were immunoprecipitated from the indicated EC cells and subjected to western blot to detect the ADMA signal after PRMT3 overexpression or knockdown. E) Amino acid sequences near the R418 site of the METTL14 protein among different species. F) Flag proteins were immunoprecipitated from the indicated EC cells transfected with Flag‐METTL14, Flag‐METTL14^418RK^, or PRMT3, and subjected to western blot to assess the ADMA of METTL14 at the 418 R site. G) Protein stability analysis by western blot of wild‐type HEC‐1A cells and HEC‐1A cells stably expressing METTL14^418RK^ treated with CHX (20 µM) for the indicated times. H) Protein levels of PRMT3 and METTL14 by western blot of HEC‐1A cells stably expressing sh‐PRMT3 or sh‐NC treated with CHX (20 µM) for the indicated times. I) Protein levels of PRMT3, METTL14, and GPX4 in the indicated HEC‐1B cells and HEC‐1A cells. J,K) Cell viability (J) and relative MDA levels (K) of the indicated HEC‐1B cells and HEC‐1A cells. ADMA, asymmetric dimethylarginine. CHX, cycloheximide. ns, no significance; ^*^ *p* < 0.05, ^**^ *p* < 0.01, and ^***^ *p* < 0.001. Data presented as mean ± SD from at least three independent experiments.

To elucidate the mechanism by which PRMT3 regulates GPX4, we performed mass spectrometry analysis on PRMT3‐binding proteins, among which METTL14 was recognized as a protein that potentially interacts with PRMT3 (**Figure** **3**A). Subsequently, the binding of PRMT3 and METTL14 was confirmed and the presence of PRMT3‐catalyzed ADMA of METTL14 was observed (Figure 3B,C). Further analysis revealed that PRMT3 amplified the ADMA signal of METTL14, while this signal was suppressed in PRMT3‐deficient cells (Figure 3D). The high conservation of this arginine residue sequence across species suggests that this methylation may have biological functions (Figure 3E). Furthermore, we estimated the possible arginine residue location (R418) based on GPS‐MSP analysis (http://msp.biocuckoo.org/). A lysine mutation (418RK) of R418 in METTL14 resulted in a significant elimination of ADMA methylation of METTL14, suggesting that this site may be the target of arginine methylation of METTL14 by PRMT3 (Figure 3F). Additionally, this mutation shortened the half‐life of METTL14, and PRMT3 deletion prevented METTL14 protein degradation (Figure 3G,H). Besides, PRMT3 deletion rescued the downregulation of METTL14 and upregulation of GPX4 induced by PRMT3 overexpression, and PRMT3 overexpression rescued the upregulation of METTL14 and downregulation of GPX4 in PRMT3‐deficient EC cells (Figure S1F, Supporting Information). Rescue experiments further revealed the regulatory relationship between the protein expression and ferroptosis between the upstream and downstream regulatory axes of PRMT3‐METTL14‐GPX4 (Figure 3I–K; Figure S1G, Supporting Information). Altogether, PRMT3 promotes arginine methylation of METTL14 and epigenetically regulates it, mediating the inhibition of cellular ferroptosis.

### 2.4 m^6^A Methylation of GPX4 mRNA by the METTL14‐YTHDF2‐Dependent Pathway

**Figure 4:**
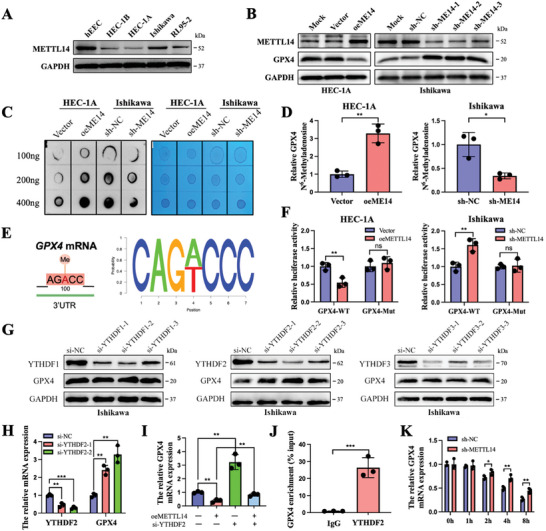
m^6^A methylation of GPX4 mRNA by the METTL14‐YTHDF2‐dependent pathway. A) Protein expression of METTL14 in EC cell lines compared with normal endometrial epithelial cells. B) Protein levels of METTL14 and GPX4 in the indicated HEC‐1A cells and Ishikawa cells detected by western blot. C) m^6^A levels of total RNAs from METTL14‐overexpressing HEC‐1A cells and METTL14‐knockdown Ishikawa cells were detected by m^6^A dot blot. The intensity of the dot blot represents the level of m^6^A modification (left), and methylene blue staining was used to detect sample loading (right). D) MeRIP‐qPCR validation of m^6^A levels of GPX4 upon METTL14 overexpression or knockdown. E) Predicted m^6^A motif on GPX4 by the SRAMP program. A luciferase reporter gene containing wild‐type or mutant (A‐to‐T mutation) GPX4 was created for subsequent assays. F) Transcript levels of wild‐type GPX4 were reduced in cells with elevated METTL14 expression, whereas deletion of METTL14 increased the luciferase activity of the wild‐type GPX4‐containing construct. G) YTHDF1/2/3 and GPX4 protein expression in Ishikawa cells transfected with si‐YTHDF1/2/3 or si‐NC. H) YTHDF2 and GPX4 mRNA expression in YTHDF2‐deficient or control Ishikawa cells. I) GPX4 expression in METTL14‐overexpressing or control Ishikawa cells in the absence or presence of YTHDF2 silencing by qRT‒PCR. J) Association of GPX4 m^6^A enrichment with YTHDF2 in Ishikawa cells detected by RIP‐qPCR. K) METTL14‐depleted or control Ishikawa cells were treated with actinomycin D, and cells were collected at respectively fixed times to determine the decay of GPX4. ns, no significance; ^*^ *p* < 0.05, ^**^ *p* < 0.01, and ^***^ *p* < 0.001. Data presented as mean ± SD from at least three independent experiments.

METTL14, as a core component of methyltransferase, can be engaged in the dynamic process of m^6^A modification, so we further investigated the m^6^A modification of GPX4 by METTL14. HEC‐1A and Ishikawa cells were selected for the experiments in this section because they were identified as cells with lower and higher METTL14 expression, respectively (**Figure** **4**A; Figure S1H, Supporting Information). Western blot and immunofluorescence assays showed that GPX4 expression was reduced in METTL14‐overexpressing cells and increased in METTL14‐deficient cells (Figure 4B; Figure S2A, Supporting Information). As shown in the results, overexpression of METTL14 dramatically enhanced the global m^6^A levels of cells and m^6^A enrichment of GPX4 mRNA (Figure 4C,D). Next, the potential m^6^A modification sites on GPX4 mRNA were identified by the SRAMP program (http://www.cuilab.cn/sramp). Luciferase reporter genes comprising wild‐type (WT) or mutant (Mut) GPX4 were generated for subsequent assays. The adenosine nucleotides in the m^6^A common sequence were substituted with thymidine in the mutant form of GPX4 (Figure 4E). The luciferase activity of the construct containing GPX4‐WT was significantly reduced upon METTL14 overexpression and enhanced upon METTL14 deletion, while the luciferase activity of GPX4‐MUT appeared to be unaffected (Figure 4F). These results revealed that GPX4 expression was controlled by METTL14‐mediated m^6^A modification.

Considering the m^6^A methylation process involves the recognition of m^6^A reader proteins, the effects of YTHDF1/2/3, as the classic m^6^A reader family, on GPX4 mRNA stability were investigated. The expression of GPX4 was found to be elevated upon YTHDF2 depletion; however, no alteration in GPX4 appeared in YTHDF1/3‐deficient cells (Figure 4G,H; Figure S2B–D). YTHDF2 deficiency eliminated the inhibitory effect on GPX4 upon METTL14 overexpression (Figure 4I). The interaction between YTHDF2 and GPX4 mRNA was confirmed by RIP‐qPCR (Figure 4J). Furthermore, both METTL14 deletion and YTHDF2 deletion significantly prolonged the half‐life of GPX4 mRNA compared to that in control cells after treatment with actinomycin D (Figure 4K; Figure S2E, Supporting Information). And the expression of METTL14 and GPX4, YTHDF2, and GPX4 were significantly negatively correlated in the TCGA‐UCEC dataset (Figure S2F,G, Supporting Information). Thus, METTL14‐mediated m^6^A modification of GPX4 promotes mRNA degradation via YTHDF2‐dependent recognition.

### 2.5 PRMT3 Expression Was Negatively Correlated with METTL14 Expression and Favorable Prognosis in EC Patients

**Figure 5:**
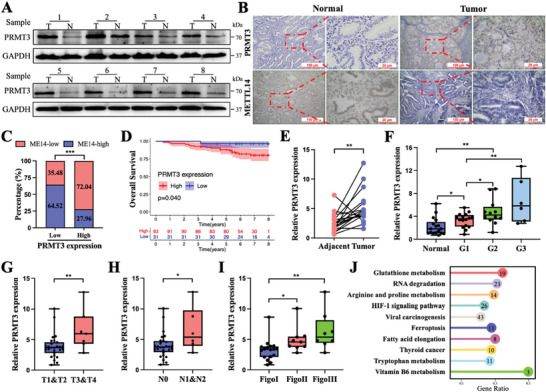
PRMT3 was negatively correlated with METTL14 expression and favorable prognosis in EC patients. A) Protein levels of PRMT3 detected by western blot in matched EC and adjacent normal tissues (*n* = 8). B) Representative immunohistochemical images of PRMT3 and METTL14 expression in EC tumor tissues and normal endometrial tissues. C) The high or low expression levels of METTL14 protein in patient tissues with high or low expression of PRMT3 classified according to IHC score were statistically analyzed. D) Kaplan‒Meier analysis of EC patients for the correlations between PRMT3 expression and overall survival (*n* = 124). E) The expression of PRMT3 in matched EC and adjacent normal tissues was measured by qRT‒PCR (*n* = 18). F) Expression of PRMT3 in normal endometrial tissues (*n* = 17) and endometrial carcinoma tissues (*n* = 34) of different pathological grades. G–I) Expression of PRMT3 in different T stages (G), N stages (H), and FIGO stages (I) of endometrial cancer tissues (*n* = 34). J) Enrichment analysis of proteins interacting with PRMT3 obtained by mass spectrometry. IHC, immunohistochemical. ns, no significance; ^*^ *p* < 0.05, ^**^ *p* < 0.01, and ^***^ *p* < 0.001. Data presented as mean ± SD from at least three independent experiments.

Further investigation and confirmation were carried out in clinical patient tissues. Western blot results showed that PRMT3 expression was higher in cancerous tissues than in adjacent normal tissues (**Figure** **5**A). As displayed in the representative images of Figure 5B, PRMT3 was highly expressed and METTL14 was expressed at low levels in tumor tissues (Figure 5B; Figure S3A,B, Supporting Information). Notably, approximately 2/3 (64.52%) of patients with low PRMT3 expression had relatively high METTL14 levels, and 72.04% of the tissues in the high PRMT3 expression group had weak METTL14 levels (Figure 5C). PRMT3 protein expression was significantly negatively correlated with METTL14 protein expression (Figure S3C, Supporting Information). In addition, the Kaplan‒Meier curves revealed that EC patients with lower PRMT3 expression had a good prognosis (Figure 5D). Consistently, qRT‒PCR data showed relatively higher levels of PRMT3 mRNA in fresh EC tumor tissues than in the corresponding adjacent normal tissues (Figure 5E). High PRMT3 expression was closely associated with late pathological grade, T stage, N stage, and FIGO stage (Figure 5F–I). The PRMT3‐interacting proteins identified by mass spectrometry were enriched in the ferroptosis pathway, further supporting our conclusions (Figure 5J). Besides, we explored the prognostic potential of PRMT3, METTL14, and GPX4 in combination. Patients with low PRMT3 expression, high METTL14 expression, and low GPX4 expression had the best prognosis. Conversely, patients with high PRMT3 expression, low METTL14 expression, and high GPX4 expression had the worst prognosis (Figure S3D, Supporting Information). These data suggest that the model composed of PRMT3, METTL14, and GPX4 could be utilized as a prognostic factor for EC patients. Overall, PRMT3 expression was upregulated in EC and might be associated with tumor progression.

### 2.6 PRMT3 Inhibition Amplified the Antitumor Effect of Anti‐PD‐1 Therapy on EC by Improving Tumor Sensitivity to Ferroptosis

Based on the report that anti‐PD‐1 therapy ultimately drives ferroptosis in cancer cells, we conducted an integrated analysis to explore the association between immunity and ferroptosis in EC. The results of the TCGA‐UCEC database indicated that the expression of recognized classical immune‐related genes is closely associated with ferroptosis‐related genes, of which GPX4 has an extremely high correlation with immune genes (Figure S4A, Supporting Information). There was a significant difference in GPX4 expression between tumor and normal tissues, and its expression in tumor tissues and paired normal tissues was also significantly different (Figure S4B–E, Supporting Information). The results of in vitro experiments confirmed that T cells can induce ferroptosis in EC, which can be partially inhibited by liproxstatin‐1. PRMT3 depletion improved cell sensitivity to T‐cell‐induced ferroptosis, which was achieved through the regulation of METTL14 by PRMT3 (Figure S5A,B, Supporting Information). As the in vitro experimental results were as expected, our next objective was to further investigate in vivo.

**Figure 6:**
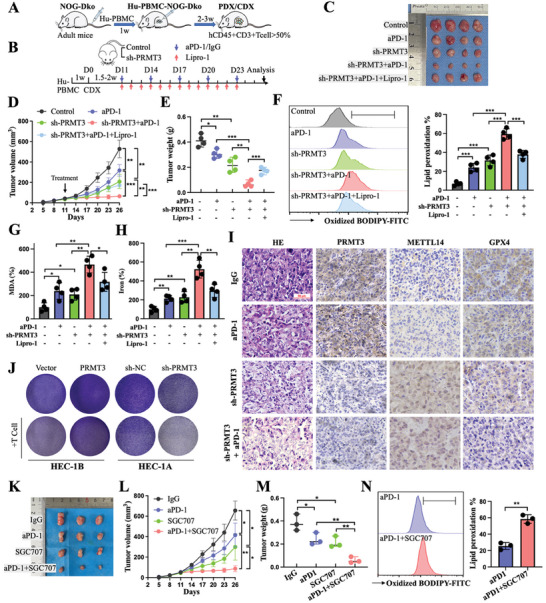
PRMT3 inhibition amplified the antitumor effect of anti‐PD‐1 therapy on EC by improving tumor sensitivity to ferroptosis. A) Schematic illustration of Hu‐NOG‐dKO PDX/CDX mouse model construction and immune cell validation. B) HEC‐1A parental and sh‐PRMT3 cells were subcutaneously inoculated into Hu‐NOG‐dKO mice models and subsequently treated with or without anti‐PD‐1, IgG, or liproxstatin‐1. C–E) Tumor images (C), volume (D), and weight (E) in different treatment groups of the mouse xenograft model. F) Levels of lipid peroxidation in tumor tissues of mice in different treatment groups. G,H) Relative levels of MDA (G) and divalent iron (H) in tumor tissues of mice in different treatment groups. I) Representative images of HE staining and IHC staining of xenograft tumors in mice of different treatment groups. J) T‐cell‐induced tumor cell killing assay performed in HEC‐1B cells from the vector and PRMT3 groups and in HEC‐1A cells from the sh‐NC and sh‐PRMT3 groups. K–M) Tumor tissue derived from endometrial cancer patients and passaged in vivo was subcutaneously inoculated into a Hu‐NOG‐dKO mouse model and subsequently treated with or without anti‐PD‐1, IgG, or SGC707. Tumor images (K), volume (L), and weight (M) in different treatment groups of mouse xenograft model. N) Levels of lipid peroxidation in tumor tissues of mice in two different treatment groups. PDX, patient‐derived xenograft. CDX, cell‐derived xenograft. IHC, immunohistochemical. ns, no significance; ^*^ *p* < 0.05, ^**^ *p* < 0.01, and ^***^ *p* < 0.001.

Infusions of human peripheral blood mononuclear cells (Hu‐PBMCs) from the murine tail vein of adult female NOG‐dKO mice were used to construct immune reconstitution models. Flow cytometry was performed to monitor the reconstitution levels of human‐derived T lymphocytes in the peripheral blood of mice 2–3 weeks later (**Figure** **6**A). The proportion of hCD45^+^mCD45^−^ cells in the peripheral blood of Hu‐PBMC NOG mice (56.7%) increased significantly at 3 weeks compared to that of control untreated NOG mice (0%), and the proportion of hCD45^+^hCD3^+^ cells in Hu‐PBMC NOG mice (3 w, 56.9%; 4 w, 80.4%) gradually increased over time (Figure S6A, Supporting Information). Moreover, the immune organs of mice, spleen, and peripheral blood, were infiltrated with human T cells at week 4 (Figure S6B, Supporting Information). The aforesaid findings indicated that the humanized mouse model was successfully established and can be employed in subsequent studies on immune checkpoint inhibitors.

We xenografted HEC‐1A‐shPRMT3 and HEC‐1A‐vector cells in the Hu‐NOG‐dKO mouse model, and mice received PD‐1 blockade or IgG treatment at the prescribed time starting on Day 11 after injection (Figure 6B). The tumor volume and weight of the mice treated with the combination of PRMT3 downregulation and anti‐PD‐1 were further reduced compared to those of the other mice (Figure 6C–E). The combination treatment group exhibited significantly higher levels of lipid peroxidation, MDA, and Fe^2+^. Liproxstatin‐1 reversed the efficacy and extent of cell death in the combination treatment group (Figure 6F–H). Moreover, the mice in the combination treatment group were observed to have the weakest GPX4 expression, the strongest 4‐HNE (a lipid peroxidation marker) expression, lower PRMT3 expression, and higher METTL14 expression (Figure 6I; Figure S6C, Supporting Information). PRMT3‐downregulated cancer cells were more likely to be killed by T cells (Figure 6J). Furthermore, we analyzed the efficacy of combined treatment with anti‐PD‐1 and a PRMT3 inhibitor (SCG707) in the PDX model (Figure S6D, Supporting Information). Slower tumor growth, lower tumor weight, and higher lipid peroxidation, MDA, and Fe2+ levels were observed in the combined treatment group than in the other groups (Figure 6K–N; Figure S6E, Supporting Information). The combination group exhibited lower GPX4 expression, higher 4‐HNE expression, higher METTL14 expression, and no alteration in cell autophagy‐related protein expression compared to the anti‐PD‐1 monotherapy group (Figures S6F and S7A, Supporting Information). Thus, blocking PRMT3 expression or PRMT3 inhibitors may enhance the benefits of anti‐PD‐1 therapy by inhibiting the resistance of cells to ferroptosis.

### 2.7 PRMT3 Downregulation Potentiated the Antitumor Efficacy of Radiotherapy and Chemotherapy in EC

**Figure 7:**
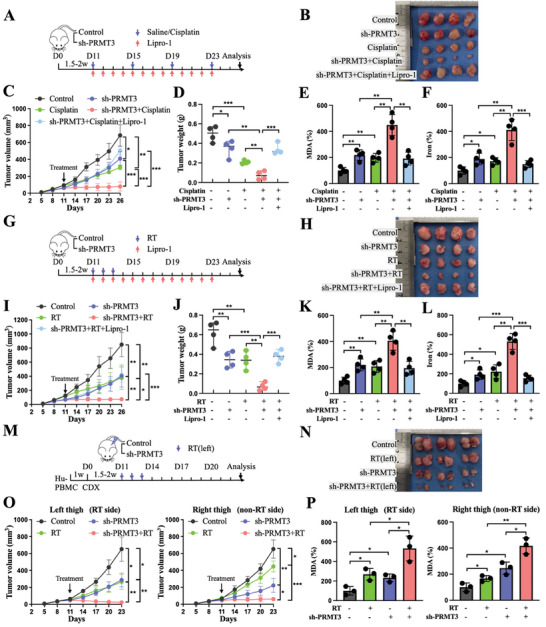
PRMT3 downregulation potentiated the antitumor efficacy of radiotherapy and chemotherapy in EC. A) HEC‐1A parental and sh‐PRMT3 cells were subcutaneously inoculated into mice xenograft models and subsequently treated with or without saline, cisplatin, or liproxstatin‐1. B–D) Tumor images (B), volume (C), and weight (D) in different treatment groups of the mouse xenograft model. E,F) Relative levels of MDA (E) and divalent iron (F) in tumor tissues of mice in different treatment groups. G) HEC‐1A parental and sh‐PRMT3 cells were subcutaneously inoculated into mice xenograft models and subsequently treated with or without radiotherapy (8 Gy) or liproxstatin‐1. H–J) Tumor images (H), volume (I), and weight (J) in different treatment groups of the mouse xenograft model. K,L) Relative levels of MDA (K) and divalent iron (L) in tumor tissues of mice in different treatment groups. M) HEC‐1A parental and sh‐PRMT3 cells were subcutaneously inoculated bilaterally into mice xenograft models and subsequently treated with or without radiotherapy (8 Gy) on the left side. N) Bilateral tumor images in different treatment groups of the mouse xenograft model. O) Tumor volume in different treatment groups of the mouse xenograft model. P) Relative levels of MDA in bilateral tumor tissues of mice in different treatment groups. RT, radiotherapy. ns, no significance; ^*^ *p* < 0.05, ^**^ *p* < 0.01, and ^***^ *p* < 0.001.

Considering the remarkable efficacy of PRMT3 knockout in enhancing immunotherapy, we then investigated whether it could have a similar impact on other therapeutic modalities, such as chemoradiotherapy and radiotherapy. Both cisplatin and radiation have been shown to accelerate ferroptosis at the cellular level. Downregulation of PRMT3 attenuated the resistance of cells to cisplatin and radiation‐induced ferroptosis by increasing METTL14 expression (Figure S7B–H, Supporting Information). Additionally, both cisplatin and radiation resulted in greater ferroptosis induction and therapeutic sensitization in the PRMT3‐deficient groups than in the other control groups, and this effect could be partially eliminated by liproxstatin‐1 (**Figure** **7**A–L). Given the existence of tumor control mechanisms beyond the radiation site, we further explored whether silencing PRMT3 in combination with radiotherapy had the potential to produce a greater abscopal effect.^[^ ^28^ ^]^ In the bilateral tumor model, radiation significantly slowed the growth of tumors on the treated side compared to the untreated control side, and there was a slight reduction in tumor volume on the nontreated side, with elevated levels of ferroptosis on both sides. Compared to those in the other three groups, bilateral tumors in the bilateral PRMT3 knockdown tumor combined with the left‐sided radiation therapy group showed stronger antitumor effects and greater ferroptosis (Figure 7M–P; Figure S8A,B, Supporting Information). These data confirmed that blocking PRMT3 improved tumor suppression in cisplatin and radiation therapy and resulted in greater activation of the abscopal effect.

## 3 Discussion

With the advancements of various therapeutic modalities, effective novel treatments in combination with classical treatments have shown extraordinary promise.^[^ ^29^, ^30^ ^]^ The immunological response of EC patients to immune checkpoint inhibitor therapy is unsatisfactory, and resistance to chemotherapy as well as radiotherapy remains an intractable challenge. As a result, developing more specific biomarkers for EC to enhance sensitivity to multiple treatment modalities is essential.

PRMTs appear to play a nonnegligible role in cancer treatment.^[^ ^31^ ^]^ Among them, PRMT3 has been reported to be involved in the regulation of glycolysis through the modification of GAPDH and LDHA.^[^ ^11^, ^13^ ^]^ Moreover, PRMT3 can recognize c‐MYC, HIF‐1α, and hnRNPA1 as substrates to affect gene expression in cancer cells.^[^ ^12^, ^14^, ^32^ ^]^ However, even so, PRMT3 is still an understudied field in EC. In the present study, integrated bioinformatic screens led to the identification of PRMT3, which was subsequently proven to promote the progression of EC.

Ferroptosis can be applied to eliminate tumors as a therapeutic strategy, and GPX4 depletion is a trigger for ferroptosis.^[^ ^33^ ^]^ Several genes and signaling pathways have been shown to achieve ferroptosis sensitivity or resistance by regulating GPX4. NFE2L1 enhances GPX4 expression to promote ferroptosis resistance, and the Wnt/β‐catenin pathway induces GPX4 expression to render tumors resistant to cisplatin‐induced ferroptosis.^[^ ^34^, ^35^ ^]^ Here, we found that PRMT3 triggers resistance to ferroptosis in EC by upregulating GPX4.

The m^6^A methylation modification of RNA can be catalyzed by methyltransferases, which have been reported to participate in the control of tumor initiation and development, drug resistance, and other processes.^[^ ^36^, ^37^, ^38^ ^]^ The current study shows that the m^6^A modification pattern distinguishes the abundance of immune infiltration in gliomas and corresponds with immunotherapy sensitivity.^[^ ^39^ ^]^ Here, we focused on methyltransferases given their irreplaceable role in disease. The results of mass spectrometry analysis showed that METTL14 is a methyltransferase that can interact with PRMT3, and this result was confirmed by immunoprecipitation assays. METTL14 exerts a tumor‐suppressive function in bladder cancer and hepatocellular cancer but promotes cancer progression and metastasis in pancreatic cancer.^[^ ^26^, ^40^, ^41^ ^]^ These seemingly contradictory results indicate the complexity of the role of m^6^A in tumor progression and highlight that METTL14 is rarely studied in EC and deserves further exploration. In our study, PRMT3 was discovered to interact with METTL14 and undergo ADMA modification of it. The results showed that PRMT3 acted as a negative regulator of METTL14 and limited its stability. Gain‐of‐function and loss‐of‐function assays additionally revealed that PRMT3‐mediated arginine methylation modification of METTL14 promoted GPX4 expression and thereby facilitated the resistance of EC cells to ferroptosis.

METTL3/14 and WTAP operate as writers to catalyze the m^6^A modification of adenosine on mRNA, and the recognition of m^6^A‐modified bases by readers is required to activate downstream regulatory processes.^[^ ^42^, ^43^ ^]^ The m^6^A methylation of lncRNA XIST catalyzed by METTL14 was recognized by YTHDF2 in colorectal cancer.^[^ ^25^ ^]^ YTHDF1 recognizes METTL14‐mediated methylation of DDB2 m^6^A in skin cancers.^[^ ^44^ ^]^ YTH has been identified as a symbolic family of m^6^A reader proteins that regulate the incoming destiny of mRNAs subject to m^6^A modification.^[^ ^45^, ^46^ ^]^ Our work shows that METTL14 downregulates GPX4 in an m^6^A‐dependent manner. Concretely speaking, GPX4 binds to the reader protein YTHDF2, which facilitates the decay of GPX4 mRNA by specifically recognizing the METTL14‐mediated consensus sequence of the m^6^A site.

In addition, Wang et al. proposed that CD8+ T cells release IFN‐γ thereby diminishing the uptake of cystine by tumor cells and resulting in ferroptosis of cancer cells. Checkpoint inhibitor therapy stimulates CD8+ T cell activation, thus inducing ferroptosis in tumors to exert antitumor effects.^[^ ^21^, ^47^ ^]^ Our work showed that PRMT3 depletion accelerated ferroptosis in tumor cells by regulating METTL14 expression. In the mouse subcutaneous transplantation tumor model, the combination of PRMT3 inhibition with anti‐PD‐1 was observed to have the most beneficial therapeutic impact and resulted in the highest level of ferroptosis in all groups. This effect could be partially antagonized by liproxstatin‐1. The results of the PDX model showed that SGC707 enhances the tumor suppressive effect of anti‐PD‐1. This finding further indicates that PRMT3 depletion enhances the responsiveness to anti‐PD‐1 treatment by sensitizing EC cells to ferroptosis. Consistently, PRMT3 inhibition driving the onset of ferroptosis leads to chemotherapy and radiotherapy sensitization and activates a more powerful abscopal effect.

Our work, of course, has some limitations. Regarding the role of ferroptosis promoters and immune checkpoint inhibitors in the treatment of malignant tumors, some scholars have different perspectives, considering that ferroptosis promoters promote the death of tumor cells as well as the occurrence of ferroptosis in immune cells, suggesting that they have a dual role. We believe that there is no contradiction and that the optimization of targeted tumor cell therapy and immunotherapy into a better fusion strategy that significantly improves benefits in patients is a breakthrough; for example, one solution is combining nanomaterials. Another, more in‐depth future validation of whether EC patients with low PRMT3 expression are more likely to benefit from multiple treatments is crucial. Moreover, PRMT may also regulate m^6^A modification and ferroptosis through other targets and pathways. PRMT5 regulates m^6^A demethylation of ALKBH5 to promote doxorubicin sensitivity in breast cancer.^[^ ^48^ ^]^ PRMT1 methylates WTAP and promotes multiple myeloma tumorigenesis by m^6^A modification of NDUFS6.^[^ ^49^ ^]^ In addition, PRMT5 inhibits ferroptosis by methylating KEAP1 in triple‐negative breast cancer, and PRMT9 suppresses ferroptosis by methylating HSPA8 in hepatocellular cancer.^[^ ^50^, ^51^ ^]^ There are complicated interaction networks in tumors, but this study mainly focuses on the PRMT3‐METTL14‐GPX4 regulatory axis in EC. In addition to this, there may be other regulatory mechanisms, which will be our future research direction. In addition, given the complexity of arginine methylation, m^6^A modification, ferroptosis, and the individual distinctions of the immune microenvironment, we should remain cautious and subsequently perform in‐depth studies in multiple biological models and clinical trials.

**Figure 8:**
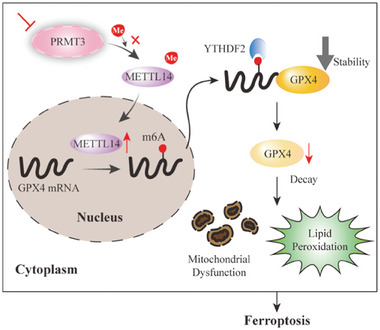
The mechanistic scheme of PRMT3 depletion to facilitate ferroptosis in EC. PRMT3 depletion fails to bind and arginine methylate METTL14, which is highly expressed and downregulates GPX4 in an m^6^A‐dependent manner, and its m^6^A site consensus sequence is recognized and bound by YTHDF2, ultimately inhibiting cellular resistance to ferroptosis and leading to therapeutic sensitization.

In summary, we identified PRMT3 as a possible biomarker for predicting unfavorable prognosis and adverse clinical progression in EC patients. We also elucidated the potential mechanism of PRMT3 in ferroptosis resistance in EC cells. PRMT3 depletion results in failure to bind and arginine methylate METTL14, which is highly expressed and downregulates GPX4 in an m^6^A‐dependent manner, ultimately inhibiting cellular resistance to ferroptosis and leading to therapeutic sensitization in EC (**Figure** **8**). Our work suggests a promising approach in which depletion of PRMT3 inhibits resistance to anti‐PD‐1, chemotherapy, and radiotherapy by promoting ferroptosis in EC.

## 4 Experimental Section

### Biological Information Analysis

The gene expression data in EC were downloaded from The Cancer Genome Atlas (TCGA) (http://cancergenome.nih.gov) database. The R software (version 4.1.3) was used for data processing. Exclusion criteria: 1) incomplete clinical data, 2) survival time less than 30 days, 3) age younger than 30 years. A total of 452 EC samples and 23 normal samples were included in the analysis.

### Cell Lines and Culture

The normal human endometrial epithelial cell line hEEC and EC cell lines HEC‐1A, HEC‐1B, Ishikawa, and RL95‐2 were provided by the Institute of Cell Biology of the Chinese Academy of Sciences (Shanghai, China). All cells were examined to determine the absence of mycoplasma contamination. hEEC cells were cultivated in a DMEM medium. HEC‐1A cells were cultivated in McCoy's 5A medium. HEC‐1B and Ishikawa cells were cultivated in MEM medium. RL95‐2 cells were grown in DMEM/F12 medium. All media were purchased from Gibco (Carlsbad, CA, USA) and supplemented with 10% fetal bovine serum (FBS, Gibco, USA) and 1% penicillin‐streptomycin (Beyotime, Shanghai, China). The incubator conditions for culturing cells were 37 °C with 5% CO2.

### Transfection

Stable overexpression of PRMT3 (oePRMT3) or matched control vector (Vector) and knockdown of PRMT3 (sh‐PRMT3) or control (sh‐NC) in human EC cell lines (GeneChem, Shanghai, China) were achieved by lentivirus as a vector. The infected cell lines were subjected to puromycin (Calbiochem, USA) for 2 weeks to screen for stable expression and validated by western blot and qRT‒PCR. Lentiviral constructs for stably overexpressing METTL14 (oeMETTL14) or matched control vector (Vector) and stably downregulating METTL14 (sh‐METTL14) or control (sh‐NC) were provided by GeneChem (Shanghai, China). Plasmids targeting GPX4 (si GPX4), YTHDF1 (si YTHDF1), YTHDF2 (si YTHDF2), YTHDF3 (si YTHDF3), and matched negative control (siNC) were constructed by GeneChem (Shanghai, China). The target sequences used for shRNA or siRNA are listed in Tables S1 and S2 (Supporting Information).

### Western Blot Analysis

RIPA containing 0.1 m PMSF was utilized to lyse cells on ice, and protein concentrations were estimated using the BCA kit. SDS‒PAGE was used to separate protein samples and the isolated proteins were then transferred to PVDF membranes (Millipore, 300 mA, 60–90 min). The protein‐covered membranes were blocked with 5% non‐fat milk and incubated with the relevant primary antibody overnight at 4 °C. The membranes and secondary antibody were coincubated at room temperature the following day. Finally, using an ECL luminous reagent, the chemiluminescent signal was generated at the matched antibody binding site on the protein membrane and visualized through a Tanon5200 imager. Experimentally relevant reagents are presented in Table S3 (Supporting Information).

### Quantitative Real Time‐PCR (qRT‒PCR)

Total RNA was isolated from the samples by using a Total RNA Kit I (Omega, R6834‐01). cDNA synthesis and PCR reactions were performed using the Reverse Transcription Kit (Roche, 0 489 703 0001) and the StepOnePlus RT‐PCR System. The primers applied were provided by Generay Biotech (Shanghai, China), and the sequences are published in Table S4 (Supporting Information). GAPDH was employed as a reference gene control, and the results were derived using the 2^−ΔΔCt^ method for quantitative analysis. The detailed procedures were identical to those depicted in earlier studies and were carried out as directed by the manufacturer.^[^ ^52^ ^]^

### CCK8 Assay

Following cell counting, separate groups of cells were cultured in 96‐well plates (4000/well) and the culture medium was removed once the designated time points were reached (0, 24, 48, 72, and 96 h). Cell Counting Kit‐8 (CCK8) solution (MCE, HY‐K0301) diluted at 10% was added to the samples and coincubated for 1.5 h. Absorbance was measured at 450 nm to assess cell viability.

### Colony Forming Assays

A total of 1000 cells were seeded into six‐well plates and cultured at 37 °C for 10–14 days. The colonies (>50 cells) were fixed with 4% paraformaldehyde and stained using 0.5% crystal violet. The crystal violet‐stained colonies were counted by three different individuals.

### Lipid Peroxidation Assessment and Cell Death Analysis

After treatment of cells or tumor tissues, the samples were stained by adding a BODIPY581/591‐C11 probe (Thermo Fisher, D3861) at a configured concentration of 10 µm and mixed incubation for 30 min. They were washed and centrifuged multiple times and resuspended in PBS, and lipid peroxidation levels were distinguished by flow cytometry. The MDA level was assessed by adopting the MDA assay‐TBA method (Nanjing Jiancheng, A003‐1), and the specific steps were meticulously adhered to as indicated by the guidelines. Cell death analysis was performed using Fixable Viability Dye eFluor (Thermo Fisher, 65‐0868‐14).

### Iron Assay

The Iron Assay Kit (Abcam, ab83366) was utilized to detect intracellular and intratissue ferrous iron levels. As per the protocols, the supernatant was obtained by centrifugation in the wake of homogenizing the tissue, or samples were obtained from cancer cells. The supernatant was blended and incubated with the assay buffer for half an hour and then incubated with an iron probe for 1 h shielded from light. Finally, the absorbance (OD 593 nm) was measured on a colorimetric microplate reader.

### Transmission Electron Microscope Imaging

EC cells from various treatment groups were gathered and fixed with 2.5% glutaraldehyde (for electron microscopy). After fixation in osmium tetroxide, the samples were dehydrated in a gradient and subsequently implanted in epoxy resin. Ultrathin sections were produced, stained, and modified, and finally, the images of the subcellular structure were monitored by a Hitachi H‐7650 transmission electron microscope (Japan).

### Coimmunoprecipitation Assay

Cell lysates were processed using A/G Magnetic Beads (MedChemExpress, HY‐K0202) as recommended by the manufacturer. The PRMT3 antibody, METTL14 antibody, Flag antibody, and IgG antibody were used for immunoprecipitation.

### Immunofluorescence

Cells inoculated on coverslips were fixed in 4% paraformaldehyde and permeabilized in 0.1% Triton. For closure, a drop of 3% BSA was applied to the coverslips for blocking, followed by a drop of diluted target antibody for incubation, and the coverslips were placed in a wet box overnight. The next day, they were treated with matching fluorescent secondary antibodies for 1 h at room temperature. The nuclei were counterstained with DAPI. Finally, imaging was performed under an FV1200 confocal microscope (Olympus, Japan). The relevant antibodies are listed in Table S3 (Supporting Information).

### m^6^A Dot Blot Assay

Purified total RNA samples were spotted onto NC membranes, which were then UV cross‐linked and closed. The membranes were then treated overnight at 4 °C with m^6^A antibody (1:1000, CST, USA), washed, and incubated for 1 h at room temperature with HRP‐coupled goat anti‐rabbit IgG (1:2000, CST, USA). After the dropwise addition of the developer, the membranes were visualized by employing the automated Tanon 5200 imaging system (Tanon, Shanghai, China). For standardization of loaded samples, 0.02% methylene blue stain was overlaid on the membranes, followed by image capture.

### MeRIP‐qPCR

Total RNA was extracted after the cells were lysed with lysis buffer. At 4 °C overnight, 1 µg of the m^6^A antibody was preconjugated to Protein G‐agarose magnetic beads. In addition, 100 µg of total RNA was incubated with the antibody‐magnetic bead conjugate in immunoprecipitation buffer (150 mm NaCl, 50 mm Tris‐HCl, and 0.5% Triton X‐100) at 4 °C for 3 h. The centrifuged precipitate was placed in 300 µL of elution buffer (5 mm Tris‐HCl, 1 mm EDTA, and 0.05% SDS). The RNA was eluted from the beads after 1.5 h of incubation at 50 °C. The RNA was extracted and purified and then subjected to qPCR analysis to determine the degree of enrichment.

### Luciferase Reporter Assay

The luciferase reporter assay was carried out as described previously.^[^ ^24^ ^]^ For reporter plasmids, wild‐type or mutant (A altered to T, Table S5, Supporting Information) target sequences, including predicted m^6^A binding sites, were synthesized separately, cloned, and inserted into the pmirGLO luciferase vector (Promega). Wild‐type or mutant F‐Luc‐GPX4 fusion reporter plasmids were transfected into cells inoculated in 6‐well plates. As indicated by the manufacturer's instructions, the luciferase activity in each well was measured 48 h after transfection by a dual‐luciferase system (Promega) to assess the regulation of GPX4 by m^6^A modification.

### RNA Stability Assay

Cells stably expressing METTL14 or control cells were cultured with 5 µg mL^−1^ actinomycin D (MedChemExpress, HY‐17559), and cells were collected after 0, 1, 2, 4, and 8 h of incubation. A total RNA kit (Omega, R6834‐01) was used to extract RNA to evaluate the half‐life of GPX4 mRNA through qRT‒PCR.

### Patients and Specimen Collection

A total of 124 EC tissues and 33 normal endometrial tissues were collected for immunohistochemistry (Table S6, Supporting Information). Another 34 fresh EC tissues were taken for PCR assays (Table S7, Supporting Information). And another 1 fresh EC tissue was taken for establishing the PDX model (Table S8, Supporting Information). All patients underwent surgery at Harbin Medical University Cancer Hospital, but no preoperative adjuvant therapy or related antitumor treatment was received by them. The study was approved by the Ethics Committee of Harbin Medical University (KY2022‐35), and each participant signed a written informed consent form.

### Hu‐NOG‐dKO Model Establishment and In Vivo Efficacy Experiments

Human peripheral blood mononuclear cells (hPBMCs) (1 × 10^7^) were injected into 6‐ to 8‐week‐old NOG‐dKO adult female mice via the tail vein, thanks to the support provided by the Beijing Charles River. One week later, patient‐derived EC tissue was transplanted to build the PDX model, as stated above, or 3 × 10^6^ logarithmically growing HEC‐1A cells were injected subcutaneously into the right hind limb region of the mice to establish the CDX model. Two to three weeks later, the growth trend of human‐derived T cells in the mouse peripheral blood was assessed by flow cytometry to determine the degree of immunological reconstitution.

Mice in the corresponding subgroups were transperitoneally administered 10 mg k^−1^g anti‐PD‐1 antibody (camrelizumab, Hengrui) or nonreactive control IgG (BioXCell, BE0093) every 3 days, 5 mg k^−1^g cisplatin (Solarbio, 15663‐27‐1) every 4 days, 10 mg k^−1^g liproxstatin‐1 (MedChemExpress, HY‐12726) every day, or 10 mg k^−1^g SGC707 (MedChemExpress, HY‐19715) every 2 days starting on day 11 of PDX/CDX model creation and continuing until the completion of the study. Throughout the process, indicators were monitored, and tumor development and weight changes were reported at regular intervals. Tumor measurements were measured by electronic calipers and tumor volume was calculated by the accompanying equation: [L × W2] × 0.5. After the experiment, tissues were collected for immunohistochemistry, western blot, and ferroptosis‐related assays. The animal experiments were approved by the Animal Care Committee of Harbin Medical University Cancer Hospital.

### Flow Cytometry

In 3rd and 4th weeks after injecting hPBMCs into NOG‐dKO mice, blood was obtained by amputation of the tail. Alternatively, mice were euthanized, blood was gathered through the heart, and spleens were gathered, crushed, and digested to a state where individual cells were suspended in the medium. For 20 min, samples were incubated with fluorescent binding antibodies. This process was followed by lysis for 10 min in a water bath with erythrocyte lysis solution. Samples were centrifuged at 4 °C to acquire the precipitate, which was then resuspended in PBS. Flow cytometry was utilized to assay samples to investigate immunological reconstitution in mice. Experimentally relevant reagents are presented in Table S3 (Supporting Information).

### Immunohistochemistry

Protein levels of PRMT3, METTL14, GPX4, and 4‐HNE in tumor tissues extracted from EC patients or mouse models were detected through immunohistochemistry. Tissue wax blocks were sectioned, deparaffinized in xylene and gradually rehydrated in gradient ethanol. Next, samples were soaked in 0.3% H_2_O_2_ to inactivate their endogenous peroxidase before being placed in an autoclave for antigen repair with EDTA, as directed in the antibody instructions. Drops of the target primary antibody were incubated overnight on the slides. Next, HRP‐conjugated secondary antibodies (Zsbio, PV‐6002, PV‐6001) and diaminobenzidine were used to complete subsequent binding and staining procedures, and typical images were acquired by visualization using a Nikon Ts2 system.

### T‐Cell‐Mediated Cancer Cell Killing

Flow cytometry was used to separate CD8+ T cells from mouse spleens, which were then placed in culture media with IL‐2 (1000 U mL^−1^). For activation of the T cells, anti‐CD3 antibody (50 ng mL^−1^) and IL‐2 were injected 1 day later. Logarithmically growing EC cells were inoculated in six‐well plates and transfected with PRMT3‐overexpressing or matched control lentivirus, as well as PRMT3 knockdown or matched control lentivirus. Activated CD8+ T cells were cocultured in a 10:1 proportion with adhered EC cells 36 h later. After 24 h, the wells were painstakingly rinsed with PBS, and the cells were fixed with paraformaldehyde and stained with crystal violet.

### PDX Establishment

Sections sampled from tumor tissues of EC patients resected during surgery were collected and cut into pieces in the ice‐cold medium. The tumor fragments were inoculated subcutaneously into the right hind limb region of 6‐ to 8‐week‐old NOD‐SCID female mice (Charles River, Beijing). Tumors underwent examination after 3 consecutive generations of propagation in mice to determine that their histological properties had not been destroyed. Growing tumors were removed and transplanted into the right flank of effectively constructed humanized‐immunized Hu‐NOG‐dKO mice.

### Statistics

Statistical analyses were conducted using the Prism 9.0, SPSS 22.0, or R 4.1.3 programs. As stated, Student's *t* test, Kaplan‒Meier curves, and Pearson's correlation analysis were performed. *p* values were calculated through three independent experiments and were presented as the mean ± SD. *p* values < 0.05 were considered statistically significant.

## Conflict of Interest

The authors declare no conflict of interest.

## Author Contributions

X.‐W.C., H.Y., and Y.‐R.W. conceived the project and designed the experimental process. X.‐W.C. and H.Y. raised funds for the project. X.‐W.C. supervised the overall project. H.Y., Y.‐R.W., and C.W. wrote and edited the manuscript with contributions from other authors. Y.Y. and Z.‐X.S. collected and organized the sample data. Y.‐R.W., C.W., X.G., Y.M., S.‐J.Z., and F.L. performed the cell experiments. Y.‐R.W., C.W., and X.G. conducted the animal experiments. Y.M., S.‐J.Z., and F.L. conducted experiments at the tissue levels. Y.‐R.W., C.W., and Y.M. analyzed and integrated the results. All authors have read and approved the article.

## Supporting information

Supporting Information

## Data Availability

The data that support the findings of this study are available from the corresponding author upon reasonable request.
