# Supplementary material for: PRMT3‐Mediated Arginine Methylation of METTL14 Promotes Malignant Progression and Treatment Resistance in Endometrial Carcinoma
Source: Adv Sci (Weinh). 2023 Nov 16;10(36):2303812. doi: 10.1002/advs.202303812 (PMC10754120; doi:10.1002/advs.202303812)
Supplement: Supplementary file 1 — Supporting Information [file ADVS-10-2303812-s001.pdf]

ADVANCED SCIENCE
Open Access

Supporting Information

for *Adv. Sci.*, DOI 10.1002/advs.202303812

PRMT3-Mediated Arginine Methylation of METTL14 Promotes Malignant Progression and Treatment Resistance in Endometrial Carcinoma

Yiru Wang, Can Wang, Xue Guan, Ying Ma, Shijie Zhang, Fei Li, Yue Yin, Zhenxing Sun, Xiuwei Chen and Hang Yin**

Fig S1

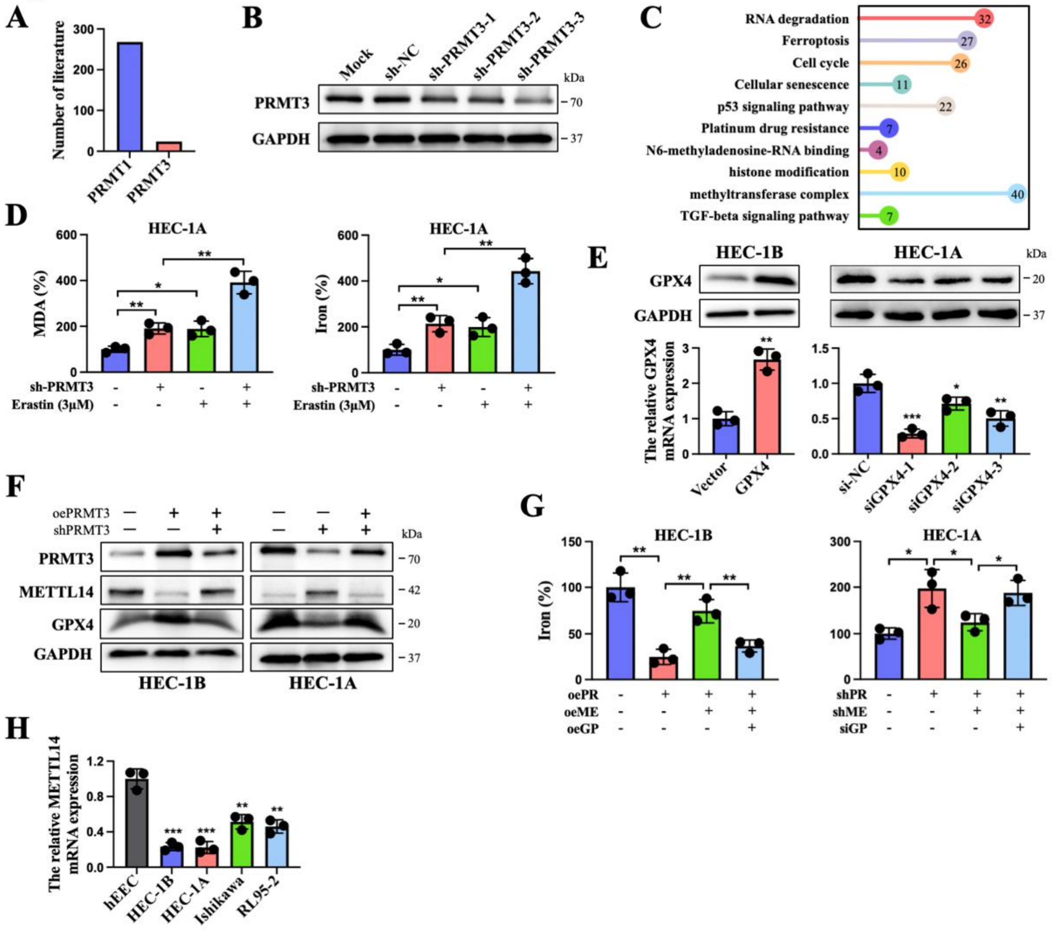

Supplementary Fig. S1. A, The number of studies that co-occurred with cancer for PRMT1 or PRMT3. **B,** Protein levels of PRMT3 in HEC-1A cells transfected with sh-PRMT3-1/2/3 or control. **C,** Functional enrichment analysis of genes co-expressed with PRMT3 in the TCGA database. **D,** Relative MDA level (**left**) and ferrous ions level (**right**) of HEC-1A cells transfected with sh-PRMT3 or treated with erastin (3 μM). **E,** Protein and mRNA levels of GPX4 in HEC-1B cells transfected with GPX4 or control and HEC-1A cells transfected with si-GPX4-1/2/3 or control. **F,** Protein levels of PRMT3, METTL14 and GPX4 in indicated HEC-1B cells and HEC-1A cells. **G,** Relative ferrous ions level of indicated HEC-1B cells and HEC-1A cells. **H,** mRNA levels of METTL14 in EC cell lines compared with normal endometrial epithelial cells. *P< 0.05, **P < 0.01, ***P < 0.001. Data presented as mean ± SD from at least three independent experiments.

Fig S2

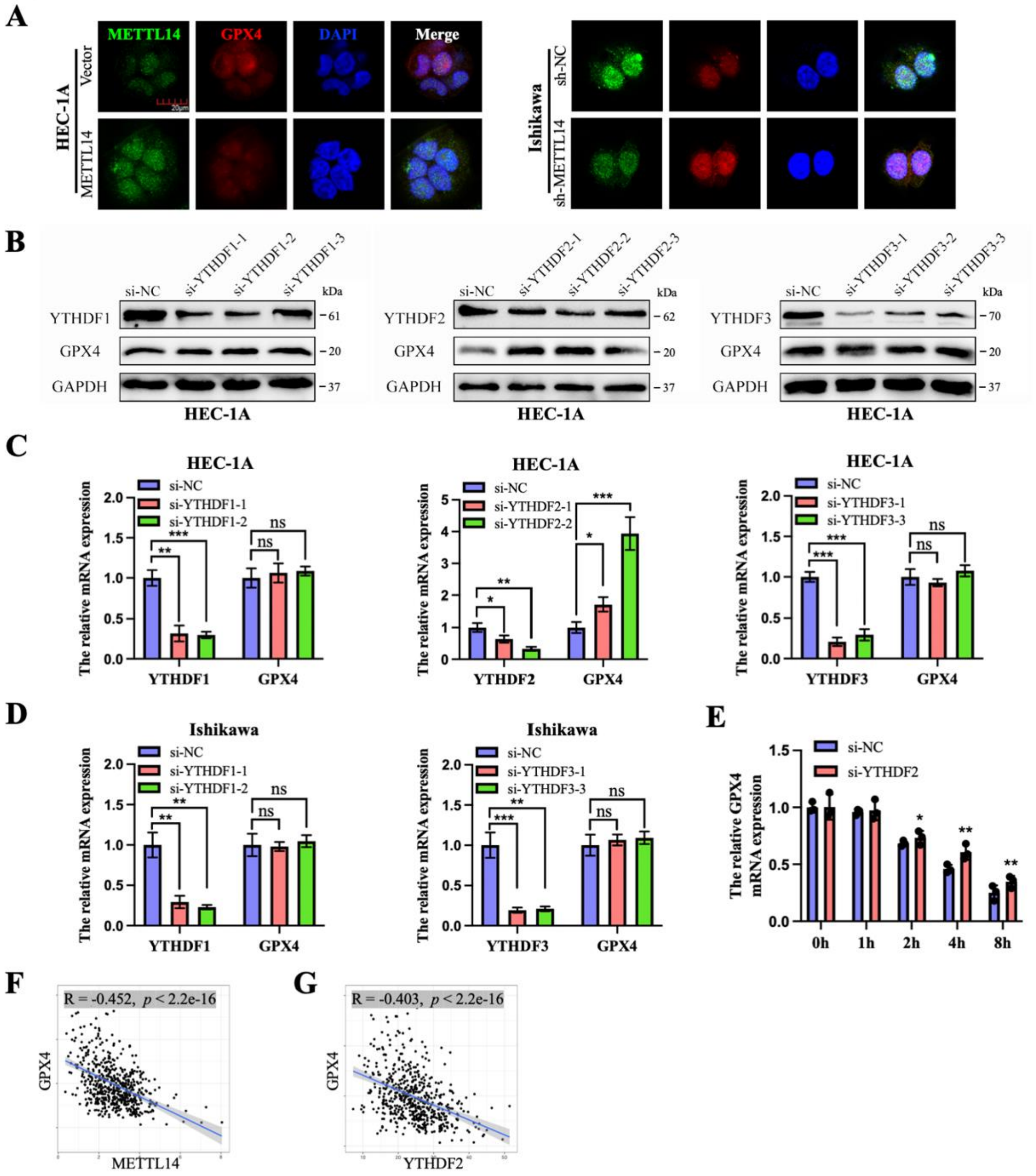

Supplementary Fig. S2. A, Protein levels of METTL14 and GPX4 in indicated HEC-1A cells and Ishikawa cells detected by immunofluorescence assay. Scale bars: 20 μm. **B,** YTHDF1/2/3 and GPX4 protein expression in HEC-1A cells transfected with si-YTHDF1/2/3 or si-NC. **C,** YTHDF1/2/3 and GPX4 mRNA expression in HEC-1A cells transfected with si-YTHDF1/2/3 or si-NC. **D,** YTHDF1/3 and GPX4 mRNA expression in Ishikawa cells transfected with si-YTHDF1/3 or si-NC. **E,** YTHDF2-knockdown or control Ishikawa cells were treated with actinomycin D, and the cells were collected

at fixed times to determine the decay of GPX4. **F,** Correlation between METTL14 and GPX4 expression in the TCGA-UCEC dataset. **G,** Correlation between YTHDF2 and GPX4 expression in the TCGA-UCEC dataset. ns, no significance; **P*< 0.05, ***P* < 0.01, ****P* < 0.001. Data presented as mean ± SD from at least three independent experiments.

Fig S3

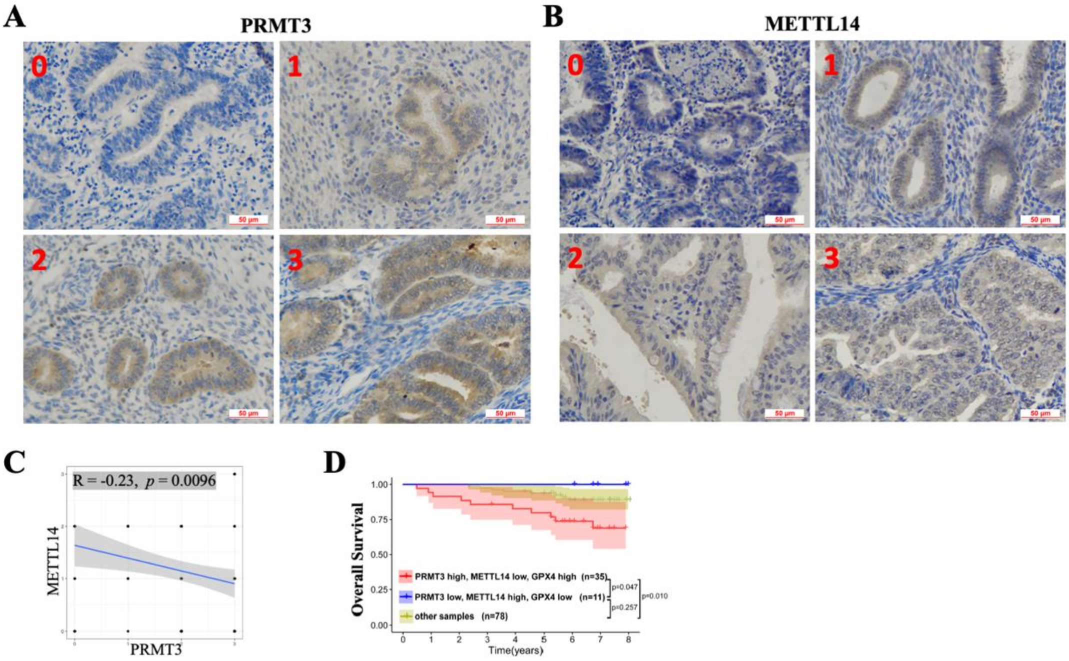

Supplementary Fig. S3. A, B, Representative immunohistochemical images of PRMT3 (**A**) and METTL14 (**B**) expression in EC tumor tissues and normal endometrium tissues graded as 0 (-, negative), 1 (+, weakly positive), 2 (++, positive) or 3 (+++, strongly positive) according to the intensity of cell staining. The tissues with scores of 2 and 3 were classified as high expression group, and the remaining tissues were viewed as low expression group. Scale bars: 50 μm. **C,** Correlation between the IHC scores of METTL14 and PRMT3 in clinical EC samples (n=124). The Pearson correlation coefficient and *p* value are indicated. **D,** Kaplan-Meier analysis of EC patients for overall survival based on the combination of the PRMT3, METTL14 and GPX4 protein expression levels (n=124).

Fig S4

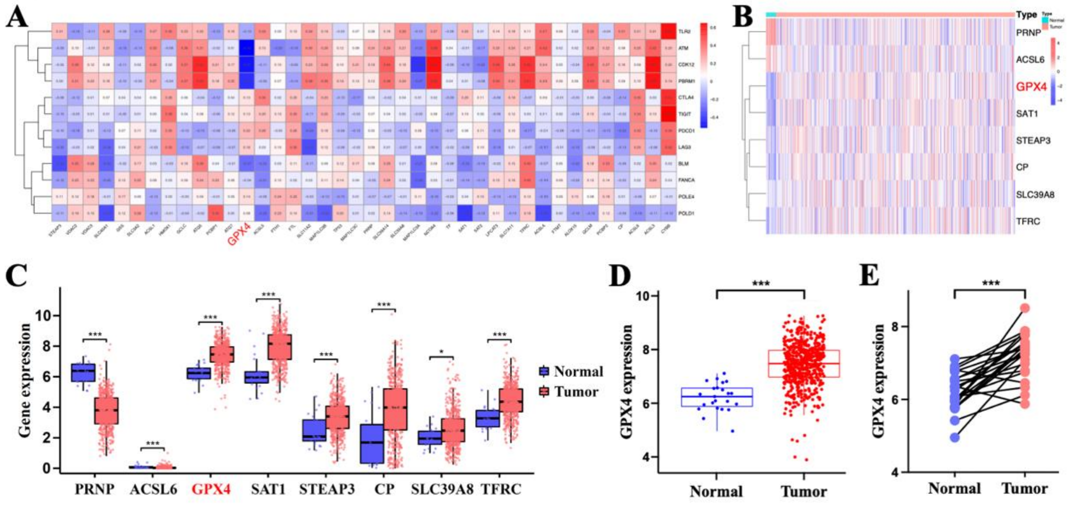

Supplementary Fig. S4. A, Co-expression analysis of recognized classical immune-related genes and ferroptosis-related genes in TCGA-UCEC database (n = 452). Red or blue color represents positive or negative correlation between the expression of the two genes. The number in the square indicates the correlation coefficient (R). **B, C,** The expressions of differentially expressed ferroptosis-related genes between tumor (n = 452) and normal (n = 23) tissues in TCGA-UCEC database. **D,** The expression of GPX4 in normal tissues (n = 23) and tumor tissues (n = 452) in TCGA-UCEC database. **E,** The expression of GPX4 in matched EC tissues and adjacent normal tissues in TCGA-UCEC database (n = 23).

Fig S5

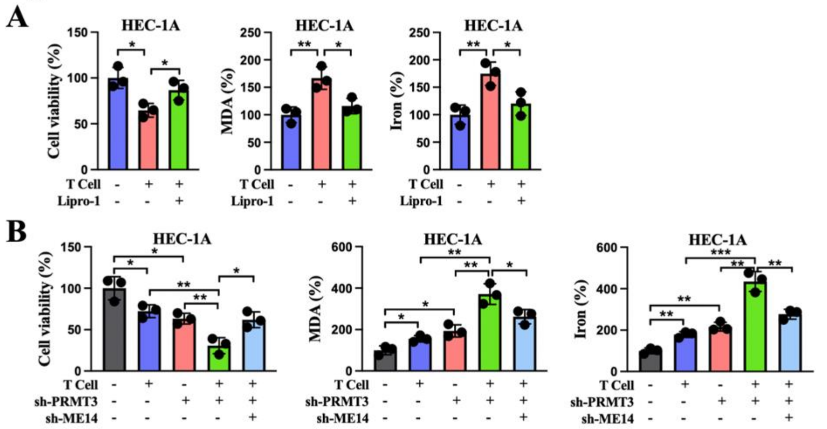

Supplementary Fig. S5. A, Cell viability (**left**), relative MDA level (**center)** and relative ferrous ions level (**right**) of HEC-1A cells co-cultured with T cells or treated with liproxstatin-1 (400 nM). **B,** Cell viability (**left**), relative MDA level (**center**) and relative ferrous ions level (**right**) of indicated HEC-1A cells. *P< 0.05, **P < 0.01, ***P < 0.001. Data presented as mean ± SD from at least three independent experiments.

Fig S6

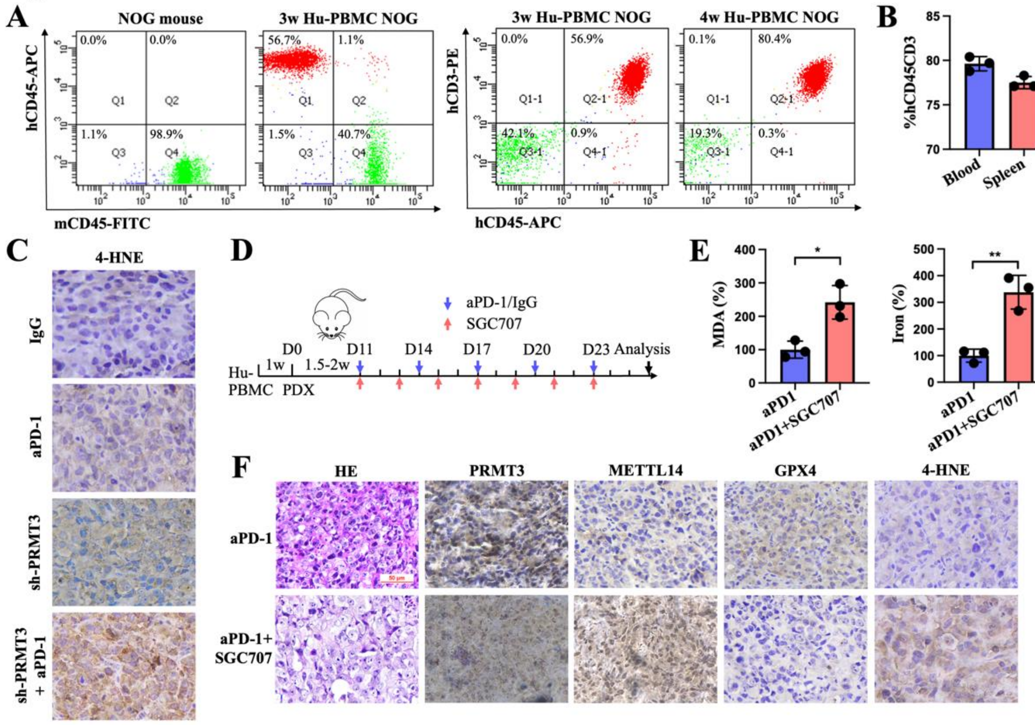

Supplementary Fig. S6. A, Levels of humanized T cells in peripheral blood of the NOG mouse model

monitored by flow cytometry. **B,** Reconstruction levels of hCD45^{+}CD3^{+} T cells in peripheral blood and spleen of Hu-NOG-dKO mice at week 4. **C.** Representative images of IHC staining of xenograft tumors in mice of different treatment groups. **D,** Scheme illustration of anti-PD-1, IgG, or SGC707 treatment regimen in Hu-NOG-dKO PDX mouse model. **E,** Relative levels of MDA (**left**) and divalent iron (**right**) in tumor tissues of mice in different treatment groups. **F,** Representative images of IHC staining of xenograft tumors in mice of different treatment groups. **P*< 0.05, ***P* < 0.01, ****P* < 0.001. Data presented as mean ± SD from at least three independent experiments.

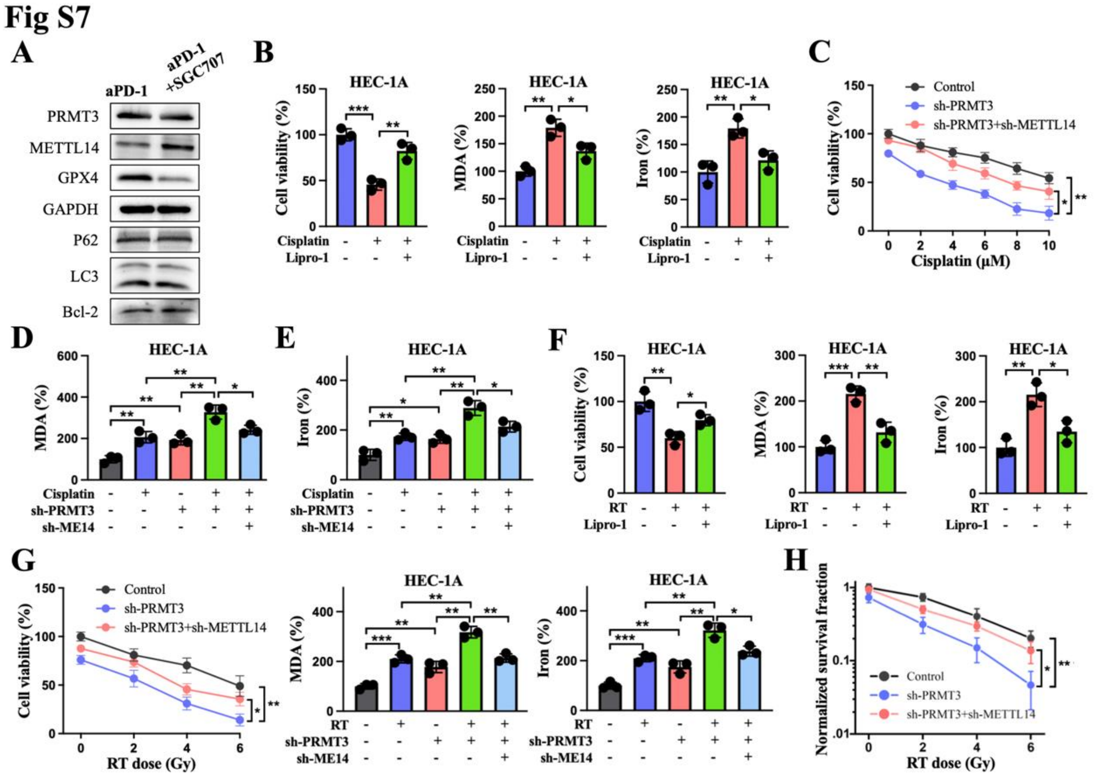

Supplementary Fig. S7. A, Protein levels of xenograft tumors in different treatment groups detected by western blot. **B,** Cell viability (**left**), relative MDA level (**Center)** and relative ferrous ions level (**right**) of HEC-1A cells treated with cisplatin (10 μM) or liproxstatin-1 (400 nM). **C,** Cell viability of indicated HEC-1A cells treated with different concentrations of cisplatin. **D, E,** Relative MDA level (**D**) and

relative ferrous ions level (**E**) of indicated HEC-1A cells. **F,** Cell viability (**left**), relative MDA level (**center)** and relative ferrous ions level (**right**) of HEC-1A cells treated with radiotherapy (6Gy) or liproxstatin-1. **G,** Cell viability (**left**), relative MDA level (**Center)** and relative ferrous ions level (**right**) of indicated HEC-1A cells. **H,** Clonogenic survival curves of the indicated HEC-1A cells exposed to different doses of radiation. **P*< 0.05, ***P* < 0.01, ****P* < 0.001. Data presented as mean ± SD from at least three independent experiments.

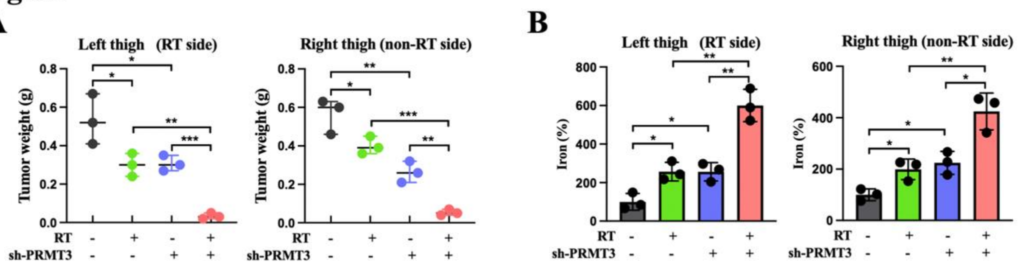

Supplementary Fig. S8. A, B, Tumor weight (**A**) and relative ferrous ions level (**B**) in bilateral tumor tissues of mice in different treatment groups.

Supplementary Table S1. The sequences of shRNAs.

shRNA Targets	Sequences
shPRMT3 sense	5′- CGTGACCCTCACGTTGAATAA -3
shMETTL14 sense	5′- GGACTTGGGATGATATTAT -3
shNC sense	5′- ACTCAAAAGGAAGTGACAAGA -3′

Supplementary Table S2. The sequences of siRNAs.

siRNA Targets	Sequences
GPX4-siRNA#1 sense	5′- GTGGATGAAGATCCAACCCAA -3
GPX4-siRNA#2 sense	5′- GTGAGGCAAGACCGAAGTAAA -3
GPX4-siRNA#3 sense	5′- AGGGAGTAACGAAGAGATCAA -3
YTHDF1-siRNA#1 sense	5′- CGCCGTCCATTGGATTTCCTT -3
YTHDF1-siRNA#2 sense	5′- TACCTGCTCTTCAGCGTCAAT -3′

YTHDF1-siRNA#3 sense	5′- CACCTACGGACAGCTCAGTAA -3′
YTHDF2-siRNA#1 sense	5′- TACTGATTAAGTCAGGATTAA -3′
YTHDF2-siRNA#2 sense	5′- CAGCCAATGAGGAAAGGGCAT -3′
YTHDF2-siRNA#3 sense	5′- ACGGTTGCATCTGCATATCCT -3′
YTHDF3-siRNA#1 sense	5′- CCAGATGGTGTATTTAGTCAA -3′
YTHDF3-siRNA#2 sense	5′- ATAACCAATTACGGCATATTC -3′
YTHDF3-siRNA#3 sense	5′- GAAGTCTGTTGTGGACTATAA -3′
NC-siRNA sense	5′- CAAGGTCGGGCAGGAAGAG -3′

Supplementary Table S3. The details of the detection antibodies for western blot analysis and Co-IP assay.

Anti-body	Manufacturer	Catalogue numbers	Dilutions
PRMT3	Abcam	ab191562	1:1000
METTL14	Abcam	ab220030	1:1000
GPX4	Abcam	ab125066	1:2000
GAPDH	Proteintech	60004-1-Ig	1:5000
Flag	Cell Signaling Technology	14793S	1:100
IgG	Cell Signaling Technology	2729S	1:100
ADMA	Cell Signaling Technology	13522S	1:1000
4-HNE	Bioss	bs-6313R	1:1000
M6A	Cell Signaling Technology	56593S	1:1000
YTHDF1	Abcam	ab220162	1:1000
YTHDF2	Abcam	ab220163	1:1000
YTHDF3	Abcam	ab220161	1:1000
Anti-mouse IgG (H+L)	Cell Signaling Technology	4408	1:200
Anti-rabbit IgG (H+L)	Cell Signaling Technology	8889	1:200
Anti-mouse HRP	Cell Signaling Technology	7076	1:2000
Anti-rabbit HRP	Cell Signaling Technology	7074	1:2000
MO-CD45	Thermo	11-0451-82	1:20
HU-CD45	Thermo	17-0459-42	1:20
HU-CD3	Thermo	12-0038-42	1:20

Supplementary Table S4. Sequences of Primer applied in Real-time Polymerase Chain Reaction.

Gene	Primer Sequences
**PRMT3**	
Forward	5’-GAACCTGCTCGTCATCTA-3’
Reverse	5’-CCATTGCCTGGTAAAGTA-3’
**METTL14**	
Forward	5’-GCATTGGTGCCGTGTTAAATAG-3’
Reverse	5’-GCATTTGGAGCAGAGGTATCA-3’
**GPX4**	
Forward	5’-CGATACGCTGAGTGTGGTTT -3’
Reverse	5’-CGGCGAACTCTTTGATCTCTT -3’
**GAPDH**	
Forward	5’-GGTGTGAACCATGAGAAGTATGA-3’
Reverse	5’-GAGTCCTTCCACGATACCAAAG-3’
**YTHDF1**	
Forward	5′-CACAGCTACAACCCGAAAGA-3′
Reverse	5′-ACTTAATGGAGCGGTGGATG-3′
**YTHDF2**	
Forward	5′-CCTCCATTGGCTTCTCCTATTC-3′
Reverse	5′-GTTGCTCAGCTGTCCATAAGA-3′
**YTHDF3**	
Forward	5′-GGGAAAGGCCCACTCTATTT-3′
Reverse	5′-AGACACCAGCATACGCATTAT-3′
**METTL14 promoter**	
Forward	AAGGCCACTCGTCTAGGAAG
Reverse	GGATAGCAGGTTCCCTTGGT

Supplementary Table S5. The sequence of wild type and mutant type of GPX4 in luciferase reporter assay.

**GPX4**	
Wild type：	CTCCACAAGTGTGTGGCCCCGCCCGAGCCCCTGCCCACG CCCTTGGAGCCTTCCACCGGCACTCATGACGGCCTGCCTGCAA ACCTGCTGGTGGGGCAGACCCGAAAATCCAGCGTGCACCCCG CCGGAGGAAGGTCCCATGGCCTGCTGGGCTTGGCTCGGCGCC CCCACCCCTGGCTACCTTGTGGGAATAAACAGACAAATTAG
Mutant type:	CTCCACAAGTGTGTGGCCCCGCCCGAGCCCCTGCCCACG CCCTTGGAGCCTTCCACCGGCACTCATGACGGCCTGCCTGCAA ACCTGCTGGTGGGGCAGTCCCGAAAATCCAGCGTGCACCCCG CCGGAGGAAGGTCCCATGGCCTGCTGGGCTTGGCTCGGCGCC CCCACCCCTGGCTACCTTGTGGGAATAAACAGACAAATTAG

Supplementary Table S6. Association between PRMT3 and clinicopathologic factors of EC patients used for immunohistochemistry.

Variables		Total (124)	%	PRMT3		*P* value
				Low(31)	High(93)	
Age(year)						0.207
	<60	86	69.4	21	51	
	≥60	38	30.6	10	42	
Histological grade						0.028
	G1	38	30.6	13	17	
	G2	54	43.5	8	31	
	G3	32	25.8	10	45	
Figo stage						0.018
	I	56	45.2	17	11	
	II	51	41.1	12	25	
	III	17	13.7	18	41	

Supplementary Table S7. The clinicopathological factors of EC patients taken for qRT-PCR assays.

Variables		Total (34)	%
Age(year)	<60	25	73.5
	≥60	9	26.5
Histological grade	G1	16	47.1
	G2	12	35.3
	G3	6	17.6
Figo stage	I	17	50.0
	II	9	26.5
	III	8	23.5
N stage	N0	6	17.6
	N1-N2	28	82.4
T stage	T1-T2	27	79.4
	T3-T4	7	20.6

Supplementary Table S8. The clinicopathological factors of the one EC patient taken for establishing the PDX model.

	Age	Histological grade	Figo stage	N stage	T stage	PRMT3 (IHC score)
Case 1	63	2	3	1	3	3
